# Metabolomic Exploration of Colorectal Cancer Through Amino Acids and Acylcarnitines Profiling of Serum Samples

**DOI:** 10.3390/cancers17030427

**Published:** 2025-01-27

**Authors:** Lucreția Avram, Dana Crișan, Radu-Cristian Moldovan, Luisa-Gabriela Bogos, Cristina-Adela Iuga, David Andraș, Sorin Crișan, Constantin Bodolea, Andrada Nemeş, Valer Donca

**Affiliations:** 1 Geriatrics—Gerontology, Department 5—Medical Specialties, Faculty of Medicine, “Iuliu Haţieganu” University of Medicine and Pharmacy Cluj-Napoca, 400012 Cluj-Napoca, Romania; avram.lucretia@umfcluj.ro (L.A.); valer.donca@umfcluj.ro (V.D.); 2 Department of Internal Medicine, 5th Medical Clinic, Faculty of Medicine, “Iuliu Hațieganu” University of Medicine and Pharmacy Cluj-Napoca, 400012 Cluj-Napoca, Romania; crisan.dana@umfcluj.ro (D.C.); sorin.crisan@umfcluj.ro (S.C.); 3 Department of Personalized Medicine and Rare Diseases, Institute of Biomedical Research—MedFuture, “Iuliu Hațieganu” University of Medicine and Pharmacy Cluj-Napoca, 400012 Cluj-Napoca, Romania; bogos.luisa@elearn.umfcluj.ro (L.-G.B.); iugac@umfcluj.ro (C.-A.I.); 4 Department of Pharmaceutical Analysis, Faculty of Pharmacy, “Iuliu Haţieganu” University of Medicine and Pharmacy, 400012 Cluj-Napoca, Romania; 5 1st Surgical Clinic, Department of General Surgery, “Iuliu Haţieganu” University of Medicine and Pharmacy, 400012 Cluj Napoca, Romania; andrasdavid88@elearn.umfcluj.ro; 6 Intensive Care Unit Department, “Iuliu Haţieganu” University of Medicine and Pharmacy, 400012 Cluj-Napoca, Romania; constantin.bodolea@umfcluj.ro (C.B.); nemes.andrada.raluca@elearn.umfcluj.ro (A.N.)

**Keywords:** colorectal cancer, amino acids, acylcarnitines, metabolomics

## Abstract

Background/Objectives: Colorectal cancer (CRC) represents one of the most prevalent forms of cancer, with high mortality rates. The aim of this study was to observe and understand the metabolic changes in CRC through targeted metabolomics. Methods: Samples collected from 58 CRC patients and 35 healthy individuals have been analyzed by liquid chromatography coupled with tandem mass spectrometry (LC-MS/MS), targeting two classes of metabolites: amino acids and acylcarnitines. Results: Statistical analysis revealed 26 significantly modified (*p*-value < 0.01; |FC| > 1.2) metabolites in CRC patients compared to the control group and 22 between colon cancer and control, whereas 8 metabolites differed only significantly between rectal cancer and healthy patients. Some of these significantly modified metabolites characterize cancer-specific adaptations, such as increased energy demand, increased tumor invasiveness, capabilities to promote amino acid synthesis, and tumor resistance against acute immune response. Moreover, receiver operator characteristic (ROC) analysis revealed that a set of two acylcarnitines (C6DC and C4-OH) can differentiate between CRC patients and healthy individuals with a high degree of confidence (AUC 0.837). Conclusions: By implementing a metabolomics approach targeting amino acids and acylcarnitines, several metabolic alterations induced by CRC have been highlighted. Even though these modifications are not specific enough to act as disease markers, they might prove useful for evaluating patient status.

## Simple Summary

Colorectal cancer represents almost 10% of all cancer cases, with high mortality rates and a high impact on patient’s quality of life. Even though screening methods help with timely diagnosis, new disease markers are needed to better understand disease progression and stratify diagnosed patients. Measurement of amino acids and acylcarnitines in colorectal cancer patients’ serum revealed several metabolic adaptations of cancer cells, which make it more aggressive and difficult to treat. Moreover, the accumulation of several acylcarnitines might prove useful for diagnosis.

## 1. Introduction

Colorectal cancer remains a leading cause of cancer-related death globally, representing approximately 9.6% of all cancer cases and standing as the second most common cause of cancer death worldwide, according to the latest GLOBOCAN estimate [1]. For instance, the annual decrease in CRC observed in the USA population has slowed down during the last decade to about 1–2%, mainly due to increased incidence in the population younger than 55 [2]. Despite advancements in screening and treatment, CRC’s mortality rates are compounded by its late diagnosis, with most cases detected in advanced stages. If detected in the early stages, the five-year survival rate for CRC can be as high as 90%, but this drops drastically to around 12% once metastasis occurs [3,4,5]. Although colonoscopy is a highly effective screening method, its invasiveness and associated patient discomfort often lead to low compliance. Non-invasive alternatives, such as the fecal occult blood test (FOBT) and fecal immunochemical test (FIT), while beneficial, generally lack sensitivity, particularly for early-stage CRC detection [6]. This underscores the urgent need for accessible, sensitive, and patient-friendly diagnostic tools capable of accurate early detection.

Some of the most promising approaches for identifying biomarkers associated with CRC are through metabolomics, offering a comprehensive snapshot of metabolic changes occurring in cancerous cells [7,8] that lead to new observations regarding the metabolic reprogramming that supports tumor proliferation and survival. CRC cells, for instance, exhibit a preference for aerobic glycolysis (the Warburg effect) over oxidative phosphorylation [9], which leads to increased glucose consumption and lactate production, fulfilling the biosynthetic requirements of cell growth and division. In addition to glucose metabolism, CRC involves dysregulated amino acid, lipid, and nucleotide metabolism. Amino acids such as tryptophan and glutamine play crucial roles in supplying nitrogen for nucleotide synthesis and serving as alternative energy sources to support tumor growth [10]. Similarly, lipid metabolism, including alterations in phosphatidylcholines and sphingomyelins, contributes to CRC progression by affecting cell membrane integrity, energy storage, and intracellular signaling pathways [11].

Recent advances in metabolomic profiling, enabled by mass spectrometry-based techniques, such as liquid chromatography-mass spectrometry (LC-MS) and gas chromatography-mass spectrometry (GC-MS), have facilitated the identification of biomarkers with high sensitivity and specificity for CRC diagnosis [12,13]. Both targeted and untargeted LC-MS and GC-MS have been extensively utilized to analyze plasma, serum, and tissue samples, revealing distinct metabolomic profiles associated with cancer stage, tumor location, and patient outcomes [14,15]. These include elevated levels of acylcarnitines, amino acids, and other intermediary metabolites, which not only reflect the tumor’s metabolic state but also hold potential as biomarkers for early CRC detection.

Among these biomarkers, acylcarnitines have garnered attention for their involvement in fatty acid oxidation and mitochondrial energy production. Altered levels of long-chain acylcarnitines in CRC patients have been linked to disruptions in energy metabolism, a hallmark of cancer cells [16,17,18]. Their metabolic relevance, coupled with their diagnostic potential, makes acylcarnitines valuable in CRC research, particularly for patient stratification based on metabolic phenotypes. Such stratification could facilitate personalized treatment approaches [11]. Additionally, metabolomics extends beyond diagnostics by identifying metabolites implicated in tumor progression, such as tryptophan, whose metabolism impacts the immune response and the tumor microenvironment [19]. Targeting these metabolic pathways offers a promising avenue for improving CRC treatment outcomes [20,21]. Furthermore, metabolomic profiling can monitor therapeutic responses by detecting metabolic shifts in response to chemotherapy or other interventions, enabling real-time adjustments to treatment plans.

In this study, a targeted metabolomics approach was employed to analyze serum samples from patients diagnosed with CRC compared to a healthy control group. The cohort included a total of 58 patients: 44 with colon cancer and 14 with rectal cancer, alongside 35 healthy individuals as controls. Serum samples were collected at the time of oncological diagnosis to investigate the potential of serum metabolomic profiles as diagnostic biomarkers for better identifying and stratifying CRC. Additionally, the relevance of specific metabolites previously reported as potential biomarkers was evaluated, aiming to validate these findings within the studied cohort and to expand knowledge for CRC diagnosis and monitoring.

## 2. Materials and Methods

### 2.1. Sample Collection

Patients were recruited from Clinical Municipal Hospital (Gastroenterology Department) and Emergency County Hospital (Surgical Department) in Cluj-Napoca, Romania. Only newly diagnosed and histopathologically confirmed cases of CRC were included, with exclusions for individuals with neuroendocrine carcinoma, malignant melanoma, non-Hodgkin’s lymphoma, gastrointestinal stromal tumors, and Lynch syndrome. Ethical approval was obtained from the institution’s Human Research Ethics Committee (Clinical Municipal Hospital; Protocol No. 292020) and adhered to the 1964 Helsinki Declaration and its amendments. Written informed consent was collected from all individuals involved.

A total of 58 patients with CRC alongside 35 healthy controls were included in this study. Samples were collected prospectively from patients diagnosed with CRC, classified by stage (0, I, II, III (locoregional CRC), and IV), together with relevant clinical information (Table 1). Colorectal cancer patients who underwent chemoradiotherapy received standard treatment protocols according to clinical guidelines, including 5-fluorouracil or capecitabine-based chemotherapy combined with radiotherapy at a total dose of 50.4 Gy administered over 28 fractions.

Venous blood samples were drawn, kept at 4 °C for a minimum of one hour for coagulation, and then centrifuged at 2500× *g* for 15 min at 4 °C. Each serum sample was immediately aliquoted and stored frozen at (−80) °C until analysis.

### 2.2. Chemicals and Reagents

Mass spectrometry grade acetonitrile, acetyl chloride, and 1-butanol were purchased from Merck (Darmstadt, Germany), chromatography grade methanol was purchased from Honeywell (Charlotte, NC, USA), and ultrapure water was produced using an Elga Purelab water purification system (Elga Labwater, High Wycombe, UK).

Amino acids and acylcarnitines calibrators and quality control materials were purchased from Chromsystems (Gräfelfing, Germany). The internals standards (IS) used for quantification of the targeted analytes were stable isotope-labeled standards of amino acids and acylcarnitines (cat. no. NSK-A and NSK-B, respectively) and they have been acquired from Cambridge Isotope Laboratories (Tewksbury, MA, USA). A full IS list together with their concentrations in the extraction solution is available in Supplementary Information S1.

### 2.3. Sample Preparation and Derivatization

The extraction solution was formed of methanol, which was spiked with known amounts of isotope-labeled internal standards. The derivatization solution used in this study was made of acetyl chloride 10% in 1-butanol (*v*/*v*), prepared by slowly dripping 100 mL of acetyl chloride over 900 mL of 1-butanol on an ice bath under continuous stirring.

The sample preparation consisted of analyte extraction and subsequent derivatization. In total, 5 µL of each sample were placed in a 96-well filter plate (MultiScreen-HV, Millipore, Burlington, MA, USA), 120 µL of extraction solution (with IS) was added, and then shaken (500 rpm) at room temperature (RT) for 30 min using a Thermomixer (Eppendorf, Hamburg, Germany). Then, the filter plate was centrifuged (2200 rcf, 10 min, RT), filtering the extract to another plate. The resulting solution was evaporated under a nitrogen stream using a microplate evaporator (Organomation, Berlin, MA, USA) for 30 min at 45 °C. In total, 60 µL of derivatization solution was added over the residues, followed by 20 min incubation at 65 °C. The samples were then evaporated a second time, before reconstitution with 100 µL of mobile phase and subjected to analysis.

### 2.4. Instrumentation and Methods

Amino acids and acylcarnitines in serum were measured by flow injection analysis, using a Waters UHPLC I-Class Plus chromatograph coupled with a Waters TQ-XS triple quadrupole mass spectrometer (Waters, Milford, MA, USA), equipped with an electrospray ionization source.

The flow injection analysis (FIA-MS/MS) method was adapted after previous literature reports [22,23]. The mobile phase consisted of acetonitrile/water—80/20 (*v*/*v*), pumped using a variable flow rate program (0.00 min–0.20 mL/min; 0.19 min–0.20 mL/min; 0.20 min–0.02 mL/min; 1.25 min–0.02 mL/min; 1.26 min–0.60 mL/min; 1.7 min–0.60 mL/min; 1.71–0.61 mL/min). The injection volume was 5 µL. MS detection was performed using different modes, depending on the analytes. Multiple reaction monitoring (MRM) was used for Gly, Leu, Orn, Lys, Met, Arg, Cit, argininosuccinic acid, and their IS; Neutral loss of *m*/*z* 102 was used for the rest of the amino acids, whereas Parent Ion Scan of *m*/*z* 85 was used for free carnitine and acylcarnitines. A total of 65 metabolites were analyzed, starting from which 48 metabolite sums and ratios were calculated, as detailed in Supplementary Information S2.

### 2.5. Data Processing

Primary data processing was performed using the IonLynx module of the MassLynx (Waters, Milford, MA, USA). The data were then exported (available in Supplementary Table S1), and further statistical analysis and graphical visualization were performed with MetaboAnalyst 6.0 (available online at www.metaboanalyst.ca), accessed on 21 October 2024. After importing, the data were normalized (log10 transformed and mean-centered). Two sample *t*-tests with unequal group variance were employed for differential analysis, considering a *p*-value threshold of 0.01 as significant, with adjustment for covariates (smoking habits, alcohol consumption, diabetes, arterial hypertension, coronary heart disease, and stroke), along with a fold change of |FC| ≥ 1.2. Heatmaps were generated on *t*-test significant variables, using the Euclidian distance measure and Ward clustering method. Receiver operating characteristic (ROC) curves were generated using the Biomarker analysis module of MetaboAnalyst 6.0.

## 3. Results

Profiling of amino acids and acylcarnitines was performed on samples collected from 58 colorectal cancer patients (44 colon cancer, 14 rectum cancer), together with 35 samples coming from healthy controls. To better understand the metabolism modifications induced by cancer, several comparisons have been made.

### 3.1. Differentially Expressed Metabolites Between Colorectal Cancer vs. Control Groups

**Figure 1:**
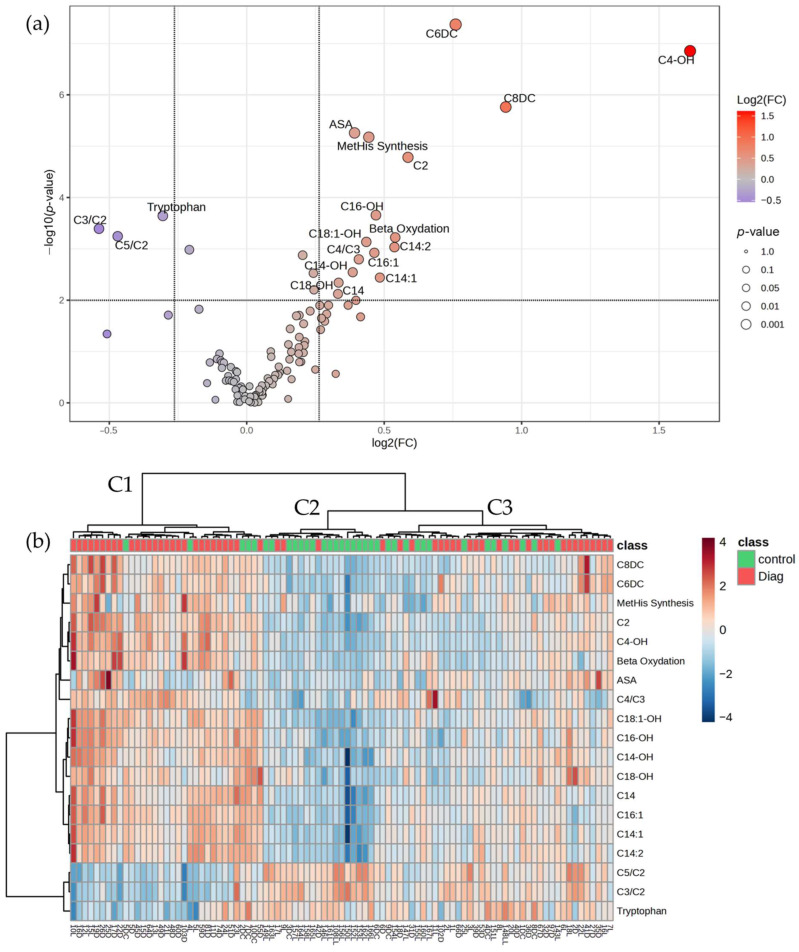
(**a**) V-plot diagram depicting the significantly variable metabolites among colorectal cancer and control groups (*p*-value < 0.01; |FC| ≥ 1.2); (**b**) Heatmap diagram of the differentially expressed metabolites between the colorectal cancer (*n* = 58) and control (*n* = 35) groups.

A set of 19 metabolites whose levels differ significantly between the two groups were identified using two-sample *t*-tests, corrected with confounding variables (see Supplementary Table S2). Among these, 16 metabolites were up-regulated (|FC| ≥ 1.2), whereas 3 were down-regulated (|FC| ≤ 1.2) when comparing the colorectal cancer group to control subjects (Figure 1a). Colorectal cancer was characterized by increased beta-oxidation, significant increases in levels of acetylcarnitine (C2), myristoylcarnitine (C14), unsaturated long-chain acylcarnitines (C14:1, C14:2, C16:1), dicarboxylic acylcarnitines (C6DC, C8DC), hydroxylated acylcarnitines (C4-OH, C14-OH, C16-OH, C18-OH, C18:1-OH), and an increased ratio of C4/C3, together with the decreased ratios of C3/C2 and C5/C2. Tryptophan was found to decrease in colorectal cancer samples, whereas the opposite was observed for argininosuccinic acid and the synthesis rate of methylhistidine.

Figure 1b presents the variation in all 19 differentially expressed metabolites across the samples. Hierarchical sample cluster analysis, conducted using the significant metabolites, highlights three clusters of samples. The first two clusters group mainly disease (C1) and control (C2) samples, with approximately 85% correct classifications. The third cluster presents a separate group within the disease set, characterized by less homogenous variation in the significantly expressed metabolites compared to C1 and C2, being comprised mostly of disease samples (65.8%).

### 3.2. Biomarker Analysis

**Figure 2:**
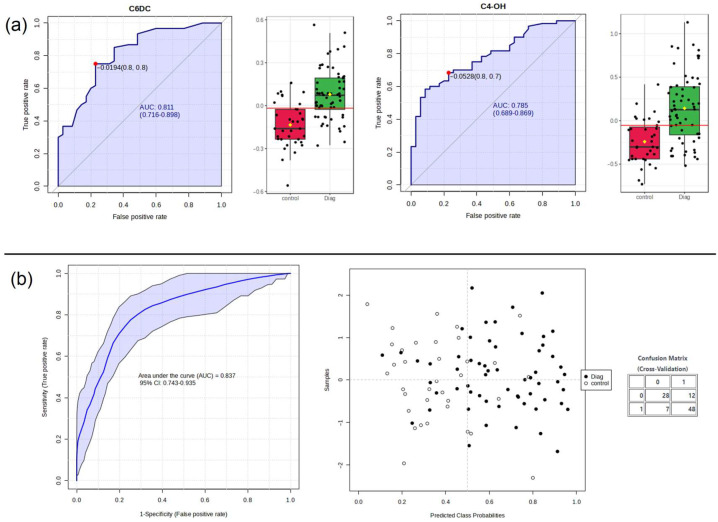
(**a**) ROC curves of the top 2 most discriminant metabolites (red line represents the cutoff value for best specificity and sensitivity); (**b**) Biomarker model created using C6DC-Carnitine and C4-OH-Carnitine as variables. ROC curve (**left**) and the average of predicted class probabilities (**right**), along with the confusion matrix.

The capability of one or multiple metabolites to correctly classify the samples to their group was assessed using the Biomarker Analysis module of Metaboanalyst 6.0, based on ROC analysis, measuring the area under the curve (AUC) as a performance assessment. AUC values for all metabolites can be found in Supplementary Table S3. Nevertheless, the top 5 most discriminant variables are C6DC (AUC 0.813), C4-OH (AUC 0.786), MetHis Synthesis (0.773), C8DC (AUC 0.772), and ASA (AUC 0.743). Moreover, a model built using two variables with the highest individual AUC (C6DC and C4-OH) managed to correctly classify 76 out of 93 samples, with an AUC of 0.837 (Figure 2).

### 3.3. Comparative Metabolomic Profiles of Colon and Rectum Cancers to the Control

#### 3.3.1. Colon Cancer vs. Control

**Figure 3:**
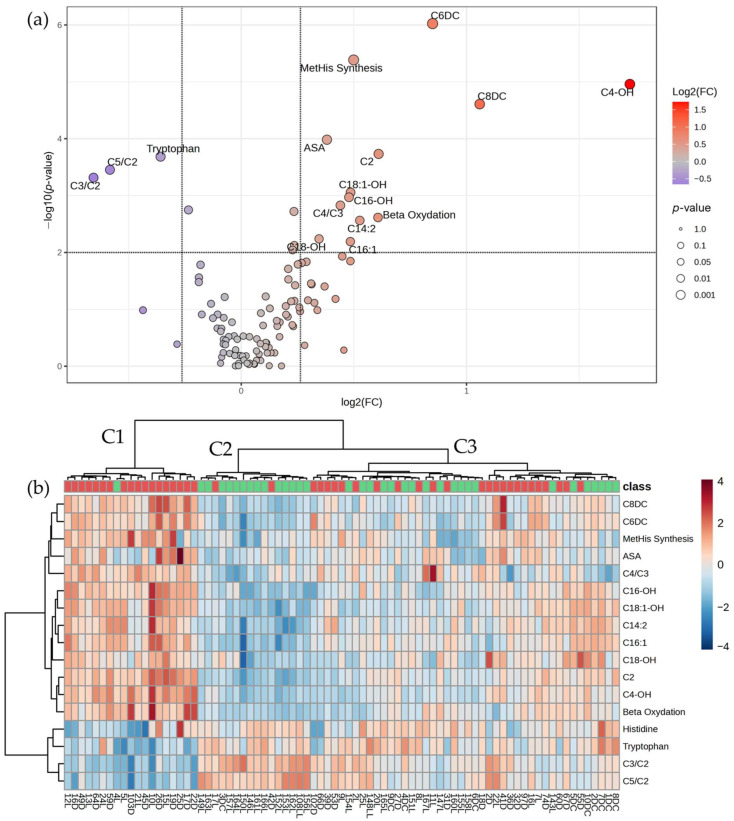
(**a**) V-plot diagram depicting the significantly variable metabolites among the colon cancer and control groups (*p*-value < 0.01; |FC| ≥ 1.2); (**b**) Heatmap diagram of the differentially expressed metabolites between the colon cancer (*n* = 44) and control (*n* = 35) groups.

A total of 17 metabolites whose levels differ significantly between the two groups were identified using a two-sample *t*-test corrected with confounding factors, with 14 metabolites up-regulated (FC ≥ 1.2), whereas 4 were down-regulated (FC ≤ 1.2) in colon cancer compared to the control (Figure 3 and Table S4). As observed for the whole colorectal cancer group, many acylcarnitines are also significantly overexpressed, such as acetylcarnitine, unsaturated long-chain acylcarnitines (C14:2, C16:1) hydroxylated acylcarnitines (C4-OH, C16-OH, C18-OH, C18:1-OH), and dicarboxylic acylcarnitines (C6DC, C8DC). The C4/C3 acylcarnitine ratio was observed to increase, whereas a decrease was recorded for C5/C2 and C3/C2 ratios. Concurrently, histidine and tryptophan showed lower levels in colon cancer patients than in control, while argininosuccinic acid, beta-oxidation, and the synthesis rate of methylhistidine were increased.

Hierarchical clustering of the samples revealed a subgroup of 19 samples (cluster 1—C1) mostly made of CRC patients with similar variations in their metabolites profile, in contrast with the second cluster of 16 samples containing a high proportion of control samples; cluster three contains samples that do not show a distinguishable pattern of metabolites.

Kendall rank correlation analysis revealed weak correlations between metabolites and colon cancer progression, with only two analytes being significantly correlated, C5-OH (correlation score 0.258; *p*-value 0.031) and C16-OH/C16 (correlation score 0.257; *p*-value 0.032) (see Supplementary Table S5).

#### 3.3.2. Rectal Cancer vs. Control

**Figure 4:**
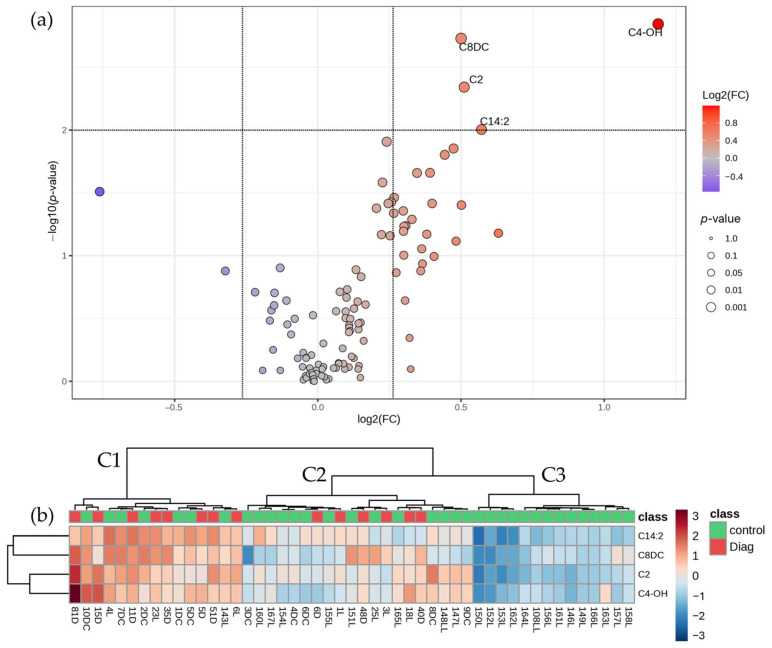
(**a**) V-plot diagram depicting the significantly variable metabolites among the rectal cancer and control groups (*p*-value < 0.01; |FC| ≥ 1.2); (**b**) Heatmap diagram of the differentially expressed metabolites between the rectal cancer (*n* = 14) and control (*n* = 35) groups.

Four metabolites whose levels differ significantly between the two groups were identified using a two-sample *t*-test adjusted for covariates, all of which are up-regulated (FC ≥ 1.2) in the rectum cancer compared to the control (Figure 4 and Table S6). Rectal cancer was characterized by fewer metabolic modifications compared to the control. Nevertheless, modifications were reflected by increased levels of several acylcarnitines, such as acetylcarnitine, 3-Hydroxybutyrylcarnitine (C4-OH), Suberylcarnitine (C8DC), and one long-chain unsaturated acylcarnitine (C14:2).

Hierarchical clustering of rectum cancer patients and controls (Figure 4b) based on the significantly modified metabolites between the two groups revealed a rather homogenous distribution of rectum cancer patients among clusters C1 and C2, with many resemblances to control samples in terms of metabolite levels.

## 4. Discussion

Colorectal cancer is characterized by profound metabolic alterations that reflect the tumors’ adaptations to increased energy and structural demands. This study investigated these changes through a detailed analysis of acylcarnitines and other metabolites involved in fatty acid β-oxidation, protein degradation, and amino acid metabolism.

Even though acylcarnitine measurements are usually linked to inborn errors in the oxidation of fatty acids, their role as diagnostic biomarkers is growing. Acylcarnitine levels reflect the energy metabolism expressed through fatty acid oxidation and the deficits in mitochondrial β-oxidation activity. The analysis performed on serum collected from 58 colorectal cancer patients revealed various significant metabolic modifications compared to the control group, both in terms of metabolic processes and metabolite levels. These alterations suggest disruptions in fatty acid oxidation, associated with the accumulation of β-oxidation intermediates.

Acetylcarnitine (C2-carnitine) is the main acylcarnitine found in human blood, playing an important role in energy production by providing acetyl groups for the synthesis of acetylcholine. Therefore, the increased energy demand of CRC is reflected by up-regulated acetylcarnitine, which was found to be significant for all three cancer vs. control comparisons. Elevated levels of C2-carnitine in CRC patient samples have also been reported by Ni et al. [24].

The increase in 3-hydroxybutyrylcarnitine (C4-OH), a metabolite formed from 3-hydroxybutyrate (3HB) (a ketone body), usually reflects fasting and ketosis [25], which is often associated with CRC. Beyond β-oxidation, 3-hydroxybutyrate emerges as a relevant marker in this context due to its dual-function ketone body, acting as both an energy source and a signaling molecule. Elevated levels of 3HB are associated with tumor invasiveness, with its ability to modulate gene expression through histone deacetylase (HDAC) inhibition [17] and suppression of the NLRP3 inflammasome [26], one of the few mechanisms that can suppress CRC metastatic growth [27].

Dicarboxylic acylcarnitines, such as C6DC (adipylcarnitine) and C8DC (suberylcarnitine), were found to be increased in CRC patients. Both metabolites can be synthesized from long-chain dicarboxylic acids by peroxisomes [28]. Nevertheless, according to Ribel-Madsen et al. [29], medium-chain dicarboxylic acylcarnitines (C6DC, C8DC, C10DC) can also be synthesized from their homologous dicarboxylic fatty acids by incomplete β-oxidation along with subsequent ω-oxidation, whereas C4DC is known to be a catabolic product of amino acid metabolism [30,31].

Several saturated and unsaturated long-chain acylcarnitines (C14, C16, C14:1, C14:2, C16:1, C18:1) showed increased levels in CRC patients. These metabolites have been previously documented as elevated in several heart conditions, type 2 diabetes, and insulin resistance, some of which have been previously correlated with CRC [11,32]. These acylcarnitines are formed starting from their homologous fatty acids, usually acquired through the diet. However, in some cases, unsaturated fatty acids can be converted from saturated ones in human cells [33].

Saturated and unsaturated long-chain hydroxylated acylcarnitines (C14-OH, C16-OH, C18-OH, C18:1-OH, C18:2-OH) have displayed increased levels in CRC patients. Much less is known regarding the biochemistry of these acylcarnitines, being observed elevated in certain genetic diseases, but also in type 2 diabetes [34,35] or cardiovascular diseases [36,37]. The accumulation of these long-chain hydroxylated acylcarnitines is usually associated with long-chain 3-hydroxy acyl-CoA dehydrogenase deficiency, which is an inborn error of metabolism. However, a recent study linked some defects in long-chain 3-hydroxy acyl-CoA dehydrogenase to the development and progression of hepatocellular carcinoma [38]. Therefore, the accumulation of these metabolites in CRC patients might be an avenue worth exploring in future research.

ROC analysis revealed that more than 10 variables performed acceptably (AUC > 0.7) when classifying patients and control subjects into their categories, while only one classified the patients with a probability above 80% (C6DC, AUC 0.811). A slight improvement was observed when building a prediction model based on two metabolites (C6DC and C4-OH), obtaining a classification probability of 83.7% (AUC 0.837). Considering the rather small sample size, these findings should be validated on a separate patient cohort. At the same time, investigation of metabolites level correlation with colon cancer progression revealed that only two metabolites significantly correlated, one hydroxylated short chain acylcarnitine (C5-OH) and one metabolite ratio (C16-OH/C16), with only weak correlation scores. The absence of more meaningful correlations between CRC stages suggests an early onset of CRC-specific metabolic alterations. A similar observation was made by Crotti et al. [19] regarding modifications in tryptophan metabolism, which could be observed as decreased even in precancerous lesions, and persisted during CRC progression.

Previous studies [18] have similarly emphasized the role of acylcarnitines in monitoring mitochondrial dysfunction and oxidative stress, providing a parallel perspective on their biological significance. Furthermore, Zhang et al. [17] highlighted the importance of peroxisomal β-oxidation as a compensatory mechanism, which may explain the accumulation of specific metabolites such as C6DC under tumor-associated metabolic stress. Two previous studies proposed acylcarnitine-based biomarker panels for differentiating between CRC patients and healthy controls [11] or between different colorectal pathologies, such as CRC and colorectal polyps [32], both including mainly long-chain acylcarnitines and presenting good sensitivities and specificities (>80%).

High levels of argininosuccinic acid are expected in colorectal cancer since almost all CRC subtypes present argininosuccinate synthase [39], an adaptation of the cancer cells to synthesize arginine starting from citrulline, with targeting arginine metabolism being an avenue explored for its potential in CRC treatment [40]. At the same time, tryptophan catabolism, reflected by its decrease in CRC patients, is an adaptation mechanism of the tumor cell against acute immune responses [19]. Tryptophan is degraded to kynurenine by indoleamine 2,3-dioxygenase 1 (IDO1), which is present in the tumor microenvironment (tumor, stromal and immune cells). IDO1 plays important roles in controlling adaptive immune responses, supporting inflammatory processes that suppress immunological response by cytotoxic T lymphocyte proliferation and activity. Moreover, several tryptophan metabolites act as agonists for aryl hydrocarbon receptor, a cytoplasmatic transcription factor, whose activation plays an important role in IDO1 expression [20,41]. Tryptophan levels have been also found to be affected by imbalanced microbiota and inflammation [20], with its decrease being proportional to the decrease in the quality of life of the CRC patients, a fact also supported by a recent study [10] that found higher plasma tryptophan levels to be associated with lower risk of CRC. Furthermore, kynurenine, a degradation product of tryptophan, is associated with immune evasion and tumor progression, offering a mechanistic explanation [41].

The methylhistidine/histidine ratio (MetHis/His), the ratio increased in CRC patients, reflects increased protein turnover and myofibrillar degradation, phenomena usually related to CRC-associated cachexia.

The CRC adaptations observed in this study reinforce previous literature reports. The cross-sectional view of metabolomic changes in CRC patients provided in this study identified several metabolites linked to tumor aggressiveness and metabolic reprogramming. Well-known metabolic adaptations of CRC such as altered metabolism of tryptophan and arginine have been emphasized in this study. Additionally, previously unrecognized or underreported metabolites in CRC have been noted. This is the case for the implications of 3HB, observed as elevated levels of 3-hydroxybutyrylcarnitine, which should be further explored in CRC considering its ability to modulate cancer suppression mechanisms. Also, this study reported other noteworthy acylcarnitines, namely C6DC and C8DC, which have not been yet documented as differentially expressed or important in differentiating CRC patients from healthy individuals in previous literature reports. These metabolites present promising capabilities to differentiate among the groups of the investigated cohort, either individually or combined in metabolite panels, and show new perspectives regarding the pathological mechanisms underlying CRC that should be further validated and explored.

The metabolic pathways previously highlighted based on the trends observed in the analyzed CRC cohort, such as fatty acid β-oxidation and tryptophan catabolism, present opportunities for therapeutic innovation. Targeting the kynurenine pathway [20] or modulating mitochondrial function [42] could provide alternative strategies for patients resistant to standard therapies or may potentially help in stratifying these patients for more personalized treatment approaches.

CRC is frequently diagnosed in advanced stages, when efficient therapeutic options are limited, with a great impact on patient outcome. The benefits of understanding metabolic modifications could influence clinical practice by enhancing cancer detection and monitoring treatment responses. Combined with current non-invasive screening tools, such as fecal immunochemical tests, it could increase their sensitivity for early-stage CRC detection. Real-time tracking changes in metabolite levels during chemotherapy or radiotherapy can also provide actionable feedback, enabling adjustments to therapeutic management and reducing toxicity.

A key advantage of this study is the analytical method applied for sample analysis and the classes of metabolites targeted as these are routinely used for screening inborn errors of metabolism. Considering the wide spread of infrastructures capable of measuring amino acids and acylcarnitines, understanding their variations in CRC could be rapidly translated into clinical practice.

Methodological differences, such as the absence of direct measurements for kynurenine and 3HB, highlight the need for future integrated studies. Incorporating these markers with genomic and proteomic data could provide new avenues for personalized diagnosis and innovative therapeutic strategies. The potential for these metabolites to predict responsiveness to chemo- and radiotherapy, as well as their association with overall survival and progression free survival, remains an open question. Longitudinal studies that integrate metabolomic data with clinical outcomes are needed to explore the prognostic and predictive value of these findings, particularly in the context of personalized treatment strategies.

The relatively small sample size of 58 CRC patients represents a limitation in terms of robustness, especially for proposing new biomarkers. Validation of these results on a larger cohort is necessary in order to draw definitive conclusions. Considering the exploratory nature of this study aimed at providing preliminary insights into CRC-associated metabolic reprogramming, the findings of this research establish an important basis for larger scale studies, involving multicenter collaborations and the inclusion of more diverse cohorts.

## 5. Conclusions

Profiling of amino acids and acylcarnitines in CRC patients’ serum clearly shows several metabolic modifications induced by cancer. The increased energy demand of cancer cells is reflected by altered mitochondrial oxidative status. The high aggressiveness of CRC is, at least in part, probably modulated at a metabolic level by several key molecules, such as 3-hydroxybutyric acid (reflected by 3-hydroxybutyrylcarnitine), argininosuccinic acid, and tryptophan, which act at different levels by promoting tumor invasiveness, finding alternative sources of amino acids, or fight against acute immune responses. Even though these metabolites are not specific only to CRC, better understanding of their variation could lead to developing fast cost effective and specific screening methods applicable to oncological diseases.

This study highlights the potential of metabolomic profiling to improve colorectal cancer (CRC) management by identifying key metabolic alterations such as elevated acylcarnitines, decreased tryptophan levels, and increased argininosuccinic acid. These findings provide a foundation for enhancing CRC diagnosis, stratification, and treatment, yet require further validation before integration into clinical practice.

Future efforts will focus on validating these results in larger multicenter cohorts, stratified by demographic and clinical factors, to enhance robustness and generalizability. Integrating metabolomics with complementary omics platforms, including genomics, proteomics, and microbiomics, will deepen the understanding of CRC biology and support the discovery of novel biomarkers and therapeutic targets. Longitudinal studies tracking metabolic shifts over time could assess the prognostic and predictive value of these changes, enabling real-time treatment adjustments and improved patient outcomes.

## Figures and Tables

**Table 1:** Clinicopathological data of the CRC patients and controls.

	CRC Patients	Control
Number	58	35
Gender (% males)	65	43
Age (years)	61 (±10)	40 (±10)
BMI (kg/m^2^)	27 (±5)	24 (±5)
Other pathologies		
Arterial hypertension (%)	56.9	8.3
Diabetes (%)	41.4	0
Coronary artery disease (%)	24.1	0
Stroke (%)	14.0	0
Lifestyle habits		
Smoking (%)	43.9	25
Alcohol consumption (%)	13.8	0
CRC stages		
0	2 (3.5%)	-
I	14 (24.1%)	-
II	19 (32.7%)	-
III	7 (12.1%)	-
IV	16 (27.6%)	-
Treatment		
Surgery	15 (25.9%)	-
Chemotherapy	5 (8.6%)	-
Radiotherapy	10 (17.2%)	-
Surgery, chemotherapy	24 (41.4%)	-
Surgery, radiotherapy	1 (1.7%)	-
Chemotherapy, radiotherapy	2 (3.5%)	-
Surgery, chemotherapy, radiotherapy	1 (1.7%)	-
One year survival (%)	85.9%	

## Data Availability

The metabolites measurements supporting this study are available in Supplementary Table S1. Further inquiries can be directed to the corresponding author.

## Supplementary Materials

The following supporting information can be downloaded at https://www.mdpi.com/article/10.3390/cancers17030427/s1: Supplementary Information S1. Concentrations of the internal standards; Supplementary Information S2. List of analyzed metabolites; Supplementary Table S1: Metabolites measurements; Supplementary Table S2: Significantly modified metabolites in colorectal cancer vs. control comparison; Table S3. Receiver Operator Characteristic analysis highlighting the variables capability to differentiate between colorectal cancer from control; Table S4. Significantly modified metabolites in colon cancer vs. control comparison; Table S5. Metabolites correlated to colon cancer stages; Table S6. Significantly modified metabolites in rectum cancer vs. control comparison.
