# Supplementary material for: Metabolomic Exploration of Colorectal Cancer Through Amino Acids and Acylcarnitines Profiling of Serum Samples
Source: Cancers (Basel). 2025 Jan 27;17(3):427. doi: 10.3390/cancers17030427 (PMC11816151; doi:10.3390/cancers17030427)
Supplement: Supplementary file 1 [file cancers-17-00427-s001.zip › Supplementary Information S1.pdf]

cancers

MDPI

Supplementary Information S1

Metabolomic Exploration of Colorectal Cancer through Amino Acids and Acylcarnitines Profiling of Serum Samples

Lucreția Avram [1,†], Dana Crișan [2,†], Radu-Cristian Moldovan [3,*], Luisa-Gabriela Bogos [3], Cristina-Adela Iuga [3,4], David Andraș [5], Sorin Crișan [2], Constantin Bodolea [6], Andrada Nemeş [6], Valer Donca [1]

Geriatrics—Gerontology, Department 5—Medical Specialties, Faculty of Medicine, "Iuliu Hațieganu" University of Medicine and Pharmacy Cluj-Napoca, 400012 Cluj-Napoca, Romania; avram.lucretia@umfcluj.ro (L.A.); valer.donca@umfcluj.ro (V.D.)

[2] Department of Internal Medicine, 5th Medical Clinic, Faculty of Medicine, "Iuliu Hațieganu" University of Medicine and Pharmacy Cluj-Napoca, 400012 Cluj-Napoca, Romania; crisan.dana@umfcluj.ro (D.C.); sorin.crisan@umfcluj.ro (S.C.)

[3] Department of Personalized Medicine and Rare Diseases, Institute of Biomedical Research—MedFuture, "Iuliu Hațieganu" University of Medicine and Pharmacy Cluj-Napoca, 400012 Cluj-Napoca, Romania; bogos.luisa@elearn.umfcluj.ro (L.-G.B.); iugac@umfcluj.ro (C.-A.I.)

[4] Department of Pharmaceutical Analysis, Faculty of Pharmacy, "Iuliu Hațieganu" University of Medicine and Pharmacy, 400012 Cluj-Napoca, Romania

[5] 1st Surgical Clinic, Department of General Surgery, "Iuliu Hațieganu" University of Medicine and Pharmacy, 400012 Cluj Napoca, Romania; andrasdavid88@elearn.umfcluj.ro

[6] Intensive Care Unit Department, "Iuliu Hațieganu" University of Medicine and Pharmacy, 400012 Cluj-Napoca, Romania; constantin.bodolea@umfcluj.ro (C.B.); nemes.andrada.raluca@elearn.umfcluj.ro (A.N.)

* Correspondence: moldovan.radu@umfcluj.ro

† These authors contributed equally to this work.

Simple Summary: Colorectal cancer represents almost 10% of all cancer cases, with high mortality rates and a high impact on patient's quality of life. Even though screening methods help with timely diagnosis, new disease markers are needed to better understand disease progression and stratify diagnosed patients. Measurement of amino acids and acylcarnitines in colorectal cancer patients' serum revealed several metabolic adaptations of cancer cells, which make it more aggressive and difficult to treat. Moreover, the accumulation of several acylcarnitines might prove useful for diagnosis.

Abstract: Background/Objectives: Colorectal cancer (CRC) represents one of the most prevalent forms of cancer, with high mortality rates. The aim of this study was to observe and understand the metabolic changes in CRC through targeted metabolomics. Methods: Samples collected from 58 CRC patients and 35 healthy individuals have been analyzed by liquid chromatography coupled with tandem mass spectrometry (LC-MS/MS), targeting two classes of metabolites: amino acids and acylcarnitines. Results: Statistical analysis revealed 26 significantly modified (p-value < 0.01; $|FC| > 1.2$) metabolites in CRC patients compared to the control group and 22 between colon cancer and control, whereas 8 metabolites differed only significantly between rectal cancer and healthy patients. Some of these significantly modified metabolites characterize cancer-specific adaptations, such as increased energy demand, increased tumor invasiveness, capabilities to promote amino acid synthesis, and tumor resistance against acute immune response. Moreover, receiver operator characteristic (ROC) analysis revealed that a set of two acylcarnitines (C6DC and C4-OH) can differentiate between CRC patients and healthy individuals with a high degree of confidence (AUC 0.837). Conclusions: By implementing a metabolomics approach targeting amino acids and acylcarnitines, several metabolic alterations induced by CRC have been highlighted. Even though these modifications are not specific enough to act as disease markers, they might prove useful for evaluating patient status.

Keywords: colorectal cancer; amino acids; acylcarnitines; metabolomics

Concentrations of the Internal standards

Table S7. Amino acid IS standards: Internal standard concentrations in stock amino acid IS solution and Working IS solution.

**Standard**	**Stock amino acids IS solution (µmol/L)**	**Working IS solution (µmol/L)**
^{15}N; 2-^{13}C-Glycine	2500	12.5
[$^{2}H_{4}$]-Alanine	500	2.5
[$^{2}H_{8}$]-Valine	500	2.5
[$^{2}H_{3}$]-Leucine	500	2.5
[$^{2}H_{3}$]-Methionine	500	2.5
[$^{2}H_{5}$]-Phenylalanine	500	2.5
[$^{2}H_{4}$]-Tyrosine	500	2.5
[$^{2}H_{3}$]-Aspartate	500	2.5
[$^{2}H_{3}$]-Glutamate	500	2.5
[$^{2}H_{2}$]-Ornithine-2HCl	500	2.5
[$^{2}H_{2}$]-Citrulline	500	2.5
[$^{2}H_{4}$]; 5-^{13}C-Arginine-HCl	500	2.5

Table S8. Free carnitine and acylcarnitine IS standards: Internal standard concentrations in stock acylcarnitine IS solution and Working IS solution.

**Standard**	**Stock acylcarnitines IS solution (µmol/L)**	**Working IS solution (µmol/L)**
[$^{2}H_{9}$]-Carnitine (free carnitine)	152.0	0.76
[$^{2}H_{3}$]-Acetylcarnitine (C2)	38	0.19
[$^{2}H_{3}$]-Propionylcarnitine (C3)	7.6	0.04
[$^{2}H_{3}$]-Butyrylcarnitine (C4)	7.6	0.04
[$^{2}H_{9}$]-Isovalerylcarnitine (C5)	7.6	0.04
[$^{2}H_{3}$]-Octanoylcarnitine (C8)	7.6	0.04
[$^{2}H_{9}$]-Myristoylcarnitine (C14)	7.6	0.04
[$^{2}H_{3}$]-Palmitoylcarnitine (C16)	15.2	0.08
