# Supplementary material for: Metabolomic Exploration of Colorectal Cancer Through Amino Acids and Acylcarnitines Profiling of Serum Samples
Source: Cancers (Basel). 2025 Jan 27;17(3):427. doi: 10.3390/cancers17030427 (PMC11816151; doi:10.3390/cancers17030427)
Supplement: Supplementary file 1 [file cancers-17-00427-s001.zip › Supplementary Information S2.pdf]

cancers

MDPI

Supplementary Information S2

Metabolomic Exploration of Colorectal Cancer through Amino Acids and Acylcarnitines Profiling of Serum Samples

Lucreția Avram [1,†], Dana Crișan [2,†], Radu-Cristian Moldovan [3,*], Luisa-Gabriela Bogos [3], Cristina-Adela Iuga [3,4], David Andraș [5], Sorin Crișan [2], Constantin Bodolea [6], Andrada Nemeş [6] and Valer Donca [1]

[1] Geriatrics—Gerontology, Department 5—Medical Specialties, Faculty of Medicine, "Iuliu Hațieganu" University of Medicine and Pharmacy Cluj-Napoca, 400012 Cluj-Napoca, Romania; avram.lucretia@umfcluj.ro (L.A.); valer.donca@umfcluj.ro (V.D.)
[2] Department of Internal Medicine, 5th Medical Clinic, Faculty of Medicine, "Iuliu Hațieganu" University of Medicine and Pharmacy Cluj-Napoca, 400012 Cluj-Napoca, Romania; crisan.dana@umfcluj.ro (D.C.); sorin.crisan@umfcluj.ro (S.C.)
[3] Department of Personalized Medicine and Rare Diseases, Institute of Biomedical Research—MedFuture, "Iuliu Hațieganu" University of Medicine and Pharmacy Cluj-Napoca, 400012 Cluj-Napoca, Romania; bogos.luisa@elearn.umfcluj.ro (L.-G.B.); iugac@umfcluj.ro (C.-A.I.)
[4] Department of Pharmaceutical Analysis, Faculty of Pharmacy, "Iuliu Hațieganu" University of Medicine and Pharmacy, 400012 Cluj-Napoca, Romania
[5] 1st Surgical Clinic, Department of General Surgery, "Iuliu Hațieganu" University of Medicine and Pharmacy, 400012 Cluj Napoca, Romania; andrasdavid88@elearn.umfcluj.ro
[6] Intensive Care Unit Department, "Iuliu Hațieganu" University of Medicine and Pharmacy, 400012 Cluj-Napoca, Romania; constantin.bodolea@umfcluj.ro (C.B.); nemes.andrada.raluca@elearn.umfcluj.ro (A.N.)
* Correspondence: moldovan.radu@umfcluj.ro
† These authors contributed equally to this work.

Simple Summary: Colorectal cancer represents almost 10% of all cancer cases, with high mortality rates and a high impact on patient's quality of life. Even though screening methods help with timely diagnosis, new disease markers are needed to better understand disease progression and stratify diagnosed patients. Measurement of amino acids and acylcarnitines in colorectal cancer patients' serum revealed several metabolic adaptations of cancer cells, which make it more aggressive and difficult to treat. Moreover, the accumulation of several acylcarnitines might prove useful for diagnosis.

Abstract: Background/Objectives: Colorectal cancer (CRC) represents one of the most prevalent forms of cancer, with high mortality rates. The aim of this study was to observe and understand the metabolic changes in CRC through targeted metabolomics. Methods: Samples collected from 58 CRC patients and 35 healthy individuals have been analyzed by liquid chromatography coupled with tandem mass spectrometry (LC-MS/MS), targeting two classes of metabolites: amino acids and acylcarnitines. Results: Statistical analysis revealed 26 significantly modified (p-value < 0.01; $|FC| > 1.2$) metabolites in CRC patients compared to the control group and 22 between colon cancer and control, whereas 8 metabolites differed only significantly between rectal cancer and healthy patients. Some of these significantly modified metabolites characterize cancer-specific adaptations, such as increased energy demand, increased tumor invasiveness, capabilities to promote amino acid synthesis, and tumor resistance against acute immune response. Moreover, receiver operator characteristic (ROC) analysis revealed that a set of two acylcarnitines (C6DC and C4-OH) can differentiate between CRC patients and healthy individuals with a high degree of confidence (AUC 0.837). Conclusions: By implementing a metabolomics approach targeting amino acids and acylcarnitines, several metabolic alterations induced by CRC have been highlighted. Even though these modifications are not specific enough to act as disease markers, they might prove useful for evaluating patient status.

Keywords: colorectal cancer; amino acids; acylcarnitines; metabolomics

Supplementary information S2 – List of measured metabolites and metabolite sums and ratios

	Metabolite	Observations
1.	Glycine	
2.	Leu&Ile	Sum of Leu and Ile
3.	Ornithine	
4.	Methionine	
5.	Citrulline	
6.	Arginine	
7.	Argininosuccinic acid	
8.	Alanine	
9.	Valine	
10.	Histidine	
11.	Methylhistidine	Sum of 1- and 3-methylhistidine
12.	Proline	
13.	Glutamine	
14.	Phenylalanine	
15.	Lysine	
16.	Serine	
17.	Threonine	
18.	Tyrosine	
19.	Tryptophan	
20.	Aspartic acid	
21.	Glutamic acid	
22.	C0-Carnitine	Free carnitine
23.	C2-Carnitine	Acetylcarnitine
24.	C3-Carnitine	Propionylcarnitine
25.	C4-Carnitine	Butyrylcarnitine
26.	C4-OH-Carnitine	3-Hydroxybutyrylcarnitine
27.	C4DC-Carnitine	Succinylcarnitine
28.	C5-Carnitine	Valerylcarnitine
29.	C5:1-Carnitine	Methylcrotonylcarnitine/Tiglylcarnitine
30.	C5-OH-Carnitine	3-Hydroxyvalerylcarnitine
31.	C5DC-Carnitine	Glutarylcarnitine
32.	C6-Carnitine	Caproylcarnitine
33.	C6DC-Carnitine	Adipylcarnitine
34.	C8-Carnitine	Octanoylcarnitine
35.	C8:1-Carnitine	Octenoylcarnitine
36.	C8DC-Carnitine	Suberylcarnitine
37.	C10-Carnitine	Decanoylcarnitine
38.	C10:1-Carnitine	Decenoylcarnitine
39.	C10:2-Carnitine	Decadienoylcarnitine
40.	C10DC-Carnitine	Decanedioylcarnitine
41.	C12-Carnitine	Lauroylcarnitine
42.	C12:1-Carnitine	Dodecenoylcarnitine
43.	C12-DC-Carnitine	Dodecanedioylcarnitine
44.	C14-Carnitine	Myristoylcarnitine

45.	C14:1-Carnitine	Tetradecenoylcarnitine
46.	C14:2-Carnitine	Tetradecadienoylcarnitine
47.	C14-OH-Carnitine	3-Hydroxy-tetradecanoylcarnitine
48.	C14-DC-Carnitine	Tetracanedioylcarnitine
49.	C16-Carnitine	Palmitoylcarnitine
50.	C16:1-Carnitine	Hexadecenoylcarnitine
51.	C16-OH-Carnitine	3-Hydroxy-hexadecanoylcarnitine
52.	C16:1-OH-Carnitine	3-Hydroxy-hexadecenoylcarnitine
53.	C16-DC-Carnitine	Hexadecanedioylcarnitine
54.	C18-Carnitine	Stearoylcarnitine
55.	C18:1-Carnitine	Octadecenoylcarnitine
56.	C18:2-Carnitine	Octadecadienylcarnitine
57.	C18-OH-Carnitine	3-Hydroxysteroylcarnitine
58.	C18:1-OH-Carnitine	3-Hydroxyoleylcarnitine
59.	C18:2-OH-Carnitine	3-Hydroxylinoleoylcarnitine
60.	C18-DC-Carnitine	Octadecanedioylcarnitine
61.	C20-Carnitine	Eicosanoylcarnitine
62.	C20-DC-Carnitine	Eicosanedioylcarnitine
63.	C22-Carnitine	Behenoylcarnitine
64.	C24&C18:1-DC-Carnitine	Lignoceroylcarnitine
65.	C26-Carnitine	Cerotoylcarnitine

	**Metabolites sums or ratios**	**Observations**
1.	Fischer Ratio	Ratio of branched-chain amino acids to aromatic amino acids - (Ile + Leu + Val) / (Phe + Trp + Tyr)
2.	GABR	Global arginine bioavailability ratio - Arg / (Orn + Cit)
3.	Glutaminase activity	Glu / Gln ratio
4.	Gly Synthesis	Gly / Ser ratio
5.	NESS/ESS	Ratio of non- essential to essential amino acids
6.	Pro/Cit	
7.	Cit Synthesis	Cit / Orn ratio
8.	NOS activity	Nitric oxide synthase activity - Cit / Arg ratio
9.	AA Sum	Sum of all amino acids
10.	Aromatic AA sum	Sum of all aromatic amino acids
11.	BCAA sum	Sum of branched-chain amino acids
12.	ESS AA sum	Sum of all essential amino acids
13.	NESS AA sum	Sum of all non-essential amino acids
14.	Glucogenic AA sum	Sum of glucogenic amino acids
15.	Ketogenic AA sum	Sum of ketogenic amino acids
16.	MetHis Synthesis	Methylhistidine synthesis (MetHis/His ratio)
17.	Orn Synthesis	Ornithine synthesis - Orn / Arg
18.	OTC defficiency	Ornithine transcarbamylase deficiency – Orn/Cit
19.	Valinemia	Val / Phe
20.	AC Sum	Sum of all acylcarnitines
21.	Beta Oxydation	(C2 + C3) / C0

22.	C2/C0	
23.	Leu/Ala	
24.	Leu/Phe	
25.	Phe/Tyr	
26.	C3/C16	
27.	C3/C2	
28.	C3DC	
29.	C3DC/C10	
30.	C4/C2	
31.	C4/C3	
32.	C5/C0	
33.	C5/C2	
34.	C5/C3	
35.	C5-OH/C0	
36.	C5-OH/C8	
37.	C5DC/C16	
38.	C5DC/C5-OH	
39.	C5DC/C8	
40.	C8/C10	
41.	C8/C2	
42.	C14:1/C12:1	
43.	C14:1/C16	
44.	C14:1/C2	
45.	C14:1/C4	
46.	C16-OH/C16	
47.	C24/C22	
48.	C26/C22	
